# EXAMINING PREDICTORS OF POOR SLEEP AMONG CAREGIVERS OF PERSONS LIVING WITH DEMENTIA

**DOI:** 10.1093/geroni/igae098.1625

**Published:** 2024-12-31

**Authors:** Darina Petrovsky, Elizabeth Luth

**Affiliations:** Duke University, Durham, North Carolina, United States; Rutgers University, New Brunswick, New Jersey, United States

## Abstract

Caregivers who struggle with sleep face increased stress and worse mental health outcomes. This study explored the connection between caregivers’ perceived caregiving burden and benefits and their sleep quality. Using data from the 2017 National Study of Caregiving and National Health and Aging Trends Study, we examined 790 caregivers of individuals with possible and probable dementia. Using time diary interviews, sleep quality was categorized as either “excellent/very good” or “good/fair/poor.” Predictors included caregiver perceived burden and benefit scales, each the summed responses to four questions (burden Cronbach alpha=0.75; benefit Cronbach alpha=0.70). We used multi-variable logistic regression, controlling for the sociodemographic characteristics of caregivers and persons with dementia, as well as relationship quality and sleep disturbance due to caregiving responsibilities. Caregivers’ mean age was 62.5 years old (SD=13). Approximately 69% were women and more than half were non-Hispanic White (58.6%). Fifty-five percent (55%) of caregivers were children and 44% reported “excellent/very good” sleep quality. In the multi-variable logistic regression model, a higher benefit of caregiving was associated with significantly higher odds of reporting “excellent/very good” sleep quality (AOR: 1.09, 95%CI: 1.01-1.17, p=0.02). Conversely, a higher burden of caregiving was associated with lower odds of reporting “excellent/very good” sleep quality (AOR: 0.88, 95%CI: 0.81-0.95, p=0.002). These relationships persisted independent of caregiver and care recipient demographic characteristics and interrupted sleep due to caregiving. Our findings highlight potential modifiable targets for future interventions—specifically, addressing perceived caregiver burden and maximizing benefits—to enhance sleep quality among caregivers of individuals living with dementia.

